# Two Nuclear Localization Signals in USP1 Mediate Nuclear Import of the USP1/UAF1 Complex

**DOI:** 10.1371/journal.pone.0038570

**Published:** 2012-06-06

**Authors:** Iraia Garcia-Santisteban, Kerman Zorroza, Jose Antonio Rodriguez

**Affiliations:** 1 Department of Genetics, Physical Anthropology and Animal Physiology, University of the Basque Country UPV/EHU, Leioa, Spain; 2 BioEF, Basque Foundation for Health Innovation and Research, Bilbao, Spain; Saint Louis University, United States of America

## Abstract

The human deubiquitinase USP1 plays important roles in cancer-related processes, such as the DNA damage response, and the maintenance of the undifferentiated state of osteosarcoma cells. USP1 deubiquitinase activity is critically regulated by its interaction with the WD40 repeat-containing protein UAF1. Inhibiting the function of the USP1/UAF1 complex sensitizes cancer cells to chemotherapy, suggesting that this complex is a relevant anticancer target. Intriguingly, whereas UAF1 has been reported to locate in the cytoplasm, USP1 is a nuclear protein, although the sequence motifs that mediate its nuclear import have not been functionally characterized. Here, we identify two nuclear localization signals (NLSs) in USP1 and show that these NLSs mediate the nuclear import of the USP1/UAF1 complex. Using a cellular relocation assay based on these results, we map the UAF1-binding site to a highly conserved 100 amino acid motif in USP1. Our data support a model in which USP1 and UAF1 form a complex in the cytoplasm that subsequently translocates to the nucleus through import mediated by USP1 NLSs. Importantly, our findings have practical implications for the development of USP1-directed therapies. First, the UAF1-interacting region of USP1 identified here might be targeted to disrupt the USP1/UAF1 interaction with therapeutic purposes. On the other hand, we describe a cellular relocation assay that can be easily implemented in a high throughput setting to search for drugs that may dissociate the USP1/UAF1 complex.

## Introduction

Ubiquitination is an important posttranslational modification that modulates the stability, localization and activity of many cellular proteins [1]. Protein ubiquitination is a dynamic and reversible process, and the removal of ubiquitin moieties from ubiquitinated substrates is catalyzed by a group of functionally related enzymes termed deubiquitinases (DUBs) [2], [3]. Nearly 100 DUBs are encoded by the human genome [4]. By deubiquitinating a variety of substrate proteins, human DUBs have been found to be involved in important cellular processes, such as cell cycle control [5], apoptosis [6], or the response to DNA damage [7], which are frequently deregulated in tumor cells.

Ubiquitin-specific protease 1 (USP1) is a DUB that plays several roles related to the DNA damage response. On one hand, USP1 contributes to regulate the Fanconi anaemia pathway through deubiquitination of FANCD2 [8], [9]. On the other hand, USP1 counteracts the monoubiquitination of PCNA and thus, prevents the recruitment of low fidelity DNA polymerases in the absence of damage [10]. Furthermore, USP1 is involved in the repair of double-strand DNA breaks through homologous recombination [11], and knockout of the USP1 gene in mice results in genomic instability [12]. Besides these DNA damage-related functions, a recent study has shown that USP1 deubiquitinates and stabilizes differentiation-inhibiting proteins of the ID (inhibitors of DNA binding) family, and thus contributes to preserve the undifferentiated state of osteosercoma cells [13].

All these data indicate that USP1 function is primarily related to nuclear processes. Consistent with these findings, USP1 has been shown to localize to the nucleus [8], [14]. However, although the amino acid sequence of USP1 bears several sequences that have been predicted to be putative nuclear localization signals (NLSs), no functional evaluation of the sequence motifs that mediate USP1 nuclear import has previously been carried out.

A precise regulation of DUB activity is critical to maintain the functional integrity of the processes controlled by these enzymes. Several regulatory mechanisms of USP1 activity have been described, including autocleavage at a diglycine motif [10], and interaction with the WD40 repeat-containing protein UAF1 (USP1-associated factor 1), also called p80 or WDR48 [15]. UAF1 has been shown to interact directly with USP1, forming a complex that contains stoichiometric amounts of both proteins [15]. UAF1 binding stabilizes USP1 and dramatically increases its catalytic activity [15]. Given the close functional relationship between USP1 and UAF1, their subcellular localization would also be expected to be closely related. Intriguingly, and in marked contrast to USP1, ectopically expressed UAF1 has been reported to be predominantly localized in the cytoplasm [16], [17]. However, the mechanisms that may modulate the nucleocytoplasmic localization of the USP1/UAF1 complex have not yet been studied.

Human DUBs are becoming increasingly recognized as potential therapeutic targets in the treatment of cancer [3], [18], [19]. The role of USP1 in processes that are frequently deregulated in tumor cells, suggests that this enzyme may represent a particularly promising target for anticancer therapy. In fact, it has been shown that USP1 gene ablation in chicken cells increases sensitivity to DNA cross-linkers [9]. Further supporting this view, a recent report has shown that inhibiting the deubiquitinase activity of the USP1/UAF1 complex sensitizes non-small cell lung cancer cells to DNA damaging agents [20]. The crucial role that UAF1 binding plays as regulator of USP1 deubiquitinating activity raises the possibility that interfering with the formation of the USP1/UAF1 complex may constitute a useful therapeutic strategy. A detailed characterization of the USP1/UAF1 interaction motifs is a necessary first step in order to explore this possibility. In this regard, previous studies have shown that a relatively large UAF1 segment, encompassing its WD repeats, mediates interaction with USP1 [15]. On the other hand, the USP1 sequences that mediate its binding to UAF1 have not yet been mapped.

Here, we identify two sequence motifs that mediate USP1 nuclear import. These motifs are one bipartite NLS, and a second monopartite NLS in the amino-terminal half of the protein. Our findings indicate that these NLSs mediate the nuclear localization of the USP1/UAF1 complex, supporting a model in which USP1 and UAF1 form a complex in the cytoplasm that subsequently relocates to the nucleus through active nuclear import mediated by USP1 NLSs. Moreover, we have mapped the UAF1-binding domain to a highly conserved 100 amino acid motif in the middle of USP1 (residues 420–520).

In addition to providing further insight into the biological consequences of the USP1/UAF1 interaction, the results presented here have practical implications for the development of the USP1/UAF1 as an anticancer target. On one hand, the UAF1-interacting region of USP1 identified here might potentially be targeted to disrupt the USP1/UAF1 interaction with therapeutic purposes. On the other hand, our findings provide the basis for a relocation assay that can be easily implemented in a high throughput setting to search for drugs that may dissociate the USP1/UAF1 complex and thus interfere with the activity of this complex in tumor cells.

## Materials and Methods

### Plasmids, Cloning Procedures, and Site-directed Mutagenesis

Mammalian expression plasmids encoding GFP-USP1 and Xpress-UAF1 were kindly provided by Dr. Rene Bernards and Dr. Jae Jung, respectively. USP1 cDNA was used as template in PCR reactions using high fidelity Pfu UltraII fusion HS DNA polymerase (Stratagene) to generate DNA fragments encoding the USP1 deletion mutants 1–672, 1–520, 1–500, 1–350, 1–285 and 1–269. These DNA fragments were cloned into pEYFP-C1 (Clontech) using KpnI and BamHI restriction sites. On the other hand, a double-stranded DNA sequence encoding USP1 NLS1 (KGNGKRKSDTEFGNMKKKVKLS) was generated by annealing two complementary oligonucleotides, and cloned upstream of GFP in the pEGFP-N1 plasmid using the HindIII and BamHI restriction sites. Subsequently, DNA fragments encoding USP1 deletion mutants 420–785, 450–785 and 420–520 were generated by PCR and cloned between the NLS1 and GFP-encoding sequences using the BamHI and PinAI restriction sites. The interstitial deletion mutant USP1 Del(420–520) was created using a two-step PCR procedure. Briefly, two separate DNA fragments, encoding USP1 sequences 1–419 and 521–785 with short overlapping sequences, were generated in a first PCR reaction. These fragments were purified, combined, and re-amplified to produce a final PCR product encoding USP1-Del(420–520), which was cloned into pEYFP-C1 using KpnI and BamHI restriction sites.

USP1 mutations were introduced using QuickChange Lightning Site-Directed Mutagenesis Kit (Stratagene).

**Figure 1:**
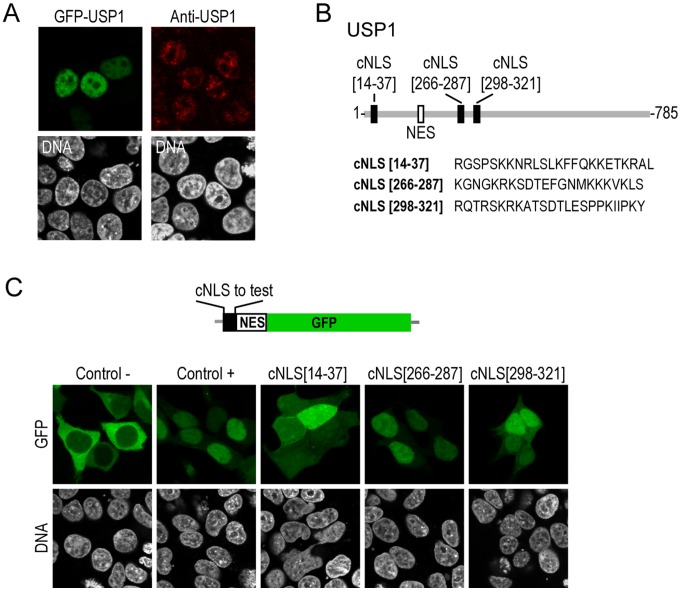
Three candidate nuclear localization signals in human USP1. A. onfocal microscopy images showing nuclear localization of GFP-(left panels), and endogenous USP1 (right panels) B. Schematic representation of human USP1 protein showing the position of three candidate NLSs (cNLSs) identified by bioinformatics analysis (black rectangles). The amino acid sequence of each cNLS is indicated below. The position of the previously reported NES is also shown (white rectangle). C. Drawing shows a schematic representation of the NES-GFP construct used in the *in vivo* nuclear import assay. Each USP1 cNLS, and the positive (PKKKRKV) and negative (PAAARAV) control sequences were cloned upstream of the NES. Confocal images show representative examples of 293T cells transfected with each of these plasmids. Cells were counterstained with Hoechst to show the nuclei (DNA panels).

The NES-GFP plasmid used in the nuclear import assay was derived from the Rev(1.4)-GFP nuclear export assay plasmid, kindly provided by Dr. Beric Henderson. We have previously described the generation of a collection of Rev(1.4)-GFP-based constructs that encode different DUB nuclear export sequences (NESs) cloned between the Rev(1.4) and the GFP moieties [14]. One of these plasmids, termed Rev(1.4)-NET22-GFP, contains a relatively strong NES (LKSLHQLLEVLLALLDKDV) identified in USP24, which was classified as 5+ according to the export assay activity score [21]. The NES-GFP plasmid was created by removing the Rev(1.4)-encoding sequence from Rev(1.4)-NET22-GFP. Subsequently, DNA sequences encoding each of USP1 candidate NLSs were inserted using HindIII and BamHI restriction sites. The SV40 NLS (PKKKRKV) and a mutant version of this sequence (PAAARAV) were similarly cloned as positive and negative controls, respectively.

**Figure 2:**
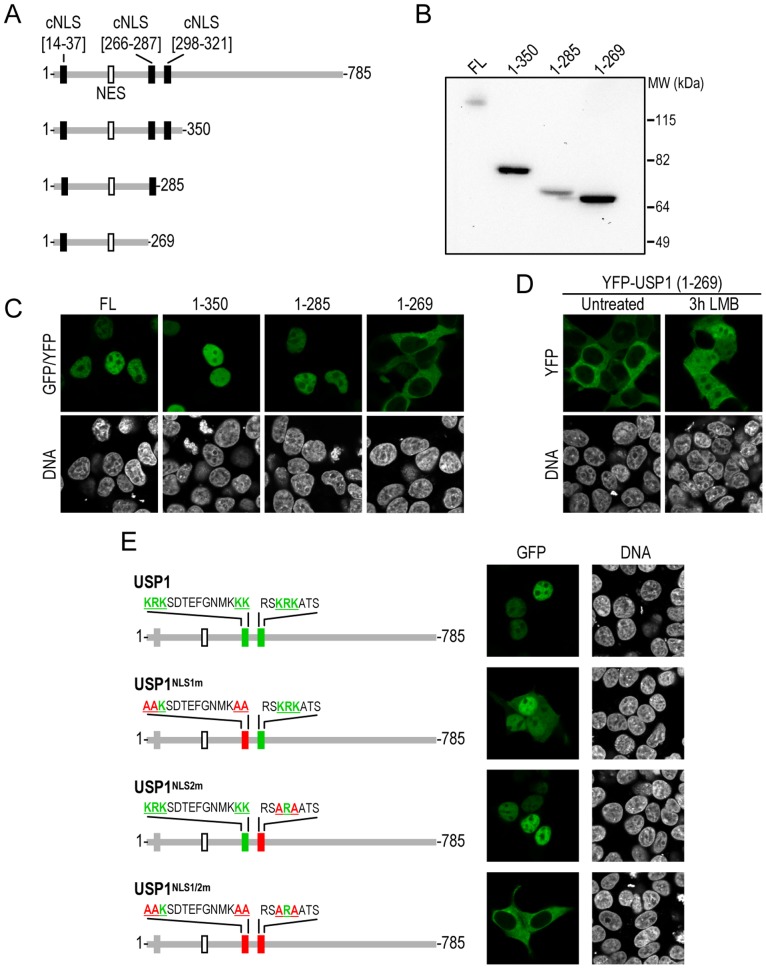
Identification of two NLSs that mediate USP1 nuclear import. A. Schematic representation of USP1 deletion mutants. The positions of the cNLS and the NES are indicated. B. Immunoblot analysis demonstrating the expression and the correct size of the different mutant proteins. C. Confocal images of 293T cells showing the nucleocytoplasmic localization of each USP1 deletion mutant. All the fragments tested were nuclear, except for the (1–269) fragment, which was exclusively located to the cytoplasm. D. Images show that inhibition of the nuclear export receptor CRM1 using leptomycin B (LMB) induces a partial relocation of YFP-USP1(1–269) to the nucleus. E. Site directed mutagenesis of USP1 NLSs in the context of the full-length protein. On the left, drawings show a schematic representation of USP1 mutants bearing alanine substitutions (red) of several basic residues (green) in NLS1, NLS2 or both. Confocal microscopy images on the right show representative examples of the nucleocytoplasmic localization of each USP1 mutant in 293T cells. Mutation of both NLSs was necessary to abrogate USP1 nuclear import. Cells were counterstained with Hoechst to show the nuclei (DNA panels).

All the new constructs generated were confirmed by DNA sequencing. The sequences of the oligonucleotides used in cloning and mutagenesis procedures are available upon request.

**Figure 3:**
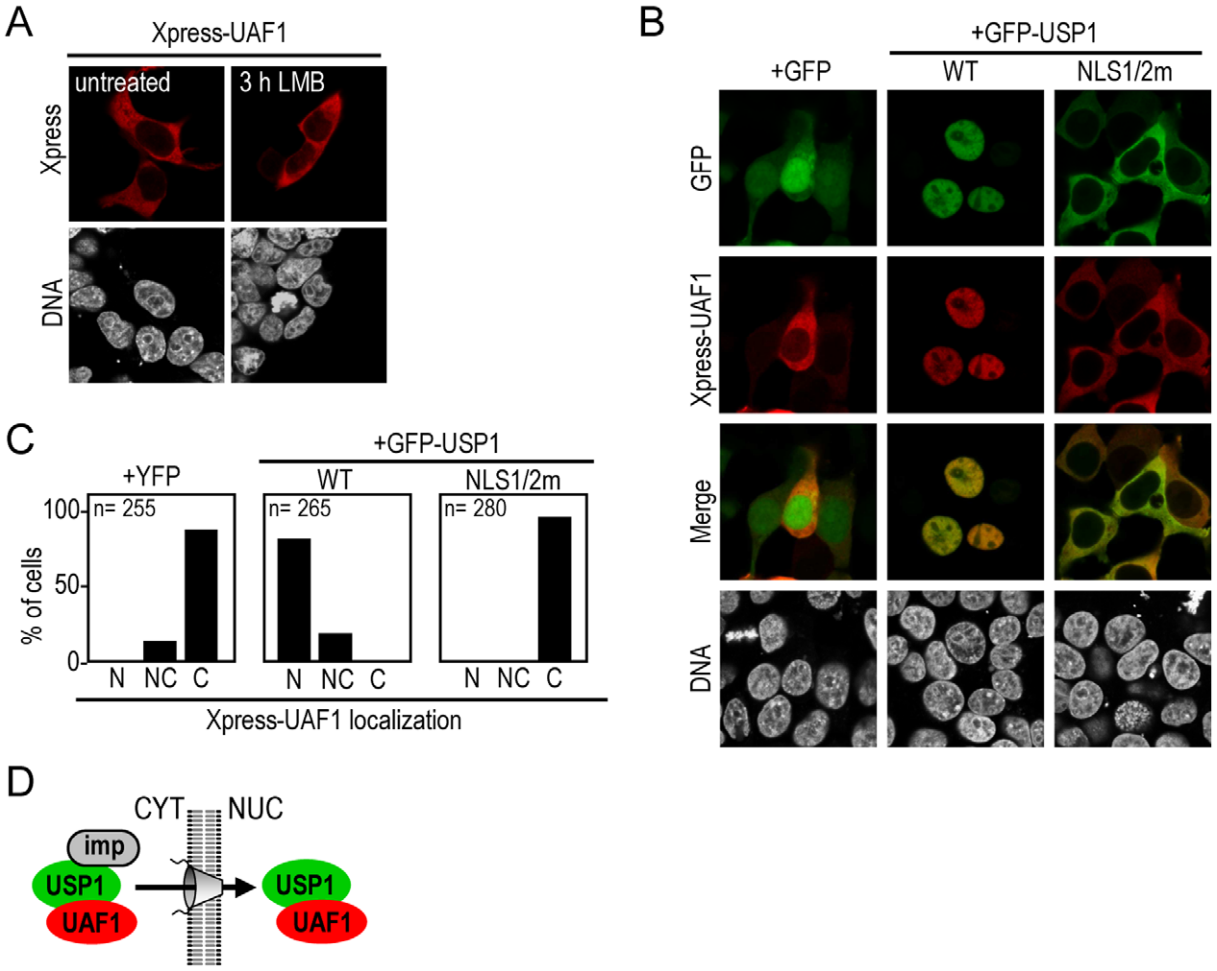
USP1 NLSs mediate nuclear import of the USP1/UAF1 complex. A. Confocal images showing that Xpress-UAF1 localizes to the cytoplasm in transfected 293T cells, and it does not relocate to the nucleus in the presence of LMB. B. Confocal images of 293T cells co-expressing Xpress-UAF1 with GFP, GFP-USP1 wild type (WT) or GFP-USP1^NLS1/2m^. Xpress-UAF1 (red) relocated to the nucleus when co-expressed with GFP-USP1 wild type, but remained in the cytoplasm when co-expressed with the import deficient mutant GFP-USP1^NLS1/2m^. Cells were counterstained with Hoechst to show the nuclei (DNA panels). C Semiquantitative analysis of Xpress-UAF1 nucleocytoplasmic distribution when co-expressed with YFP, GFP-USP1 wild type (WT) or GFP-USP1^NLS1/2m^. Graphs show the percentage of co-transfected cells showing nuclear (N), nuclear and cytoplasmic (NC) or cytoplasmic (C) localization of Xpress-UAF1. The number of cells counted in each sample (n) is indicated within the graph. D. A model illustrating the ability of USP1 to bind importins (imp) and UAF1, and thus, mediate the nuclear import of the USP1/UAF1 complex.

### Cell Culture, Transfection and Leptomycin B Treatment

Human embryonic kidney cells 293T, obtained from ATCC, were grown in Dulbecco’s modified Eagle’s medium (Invitrogen), supplemented with 10% fetal bovine serum (Invitrogen), 100 U/ml penicillin and 100 µg/ml streptomycin (Invitrogen). Twenty four hours before transfection cells were seeded onto sterile glass coverslips in 12-well trays. Transfections were carried out with X-tremeGENE 9 transfection reagent (Roche Diagnostics) following manufacturer’s protocol.

**Figure 4:**
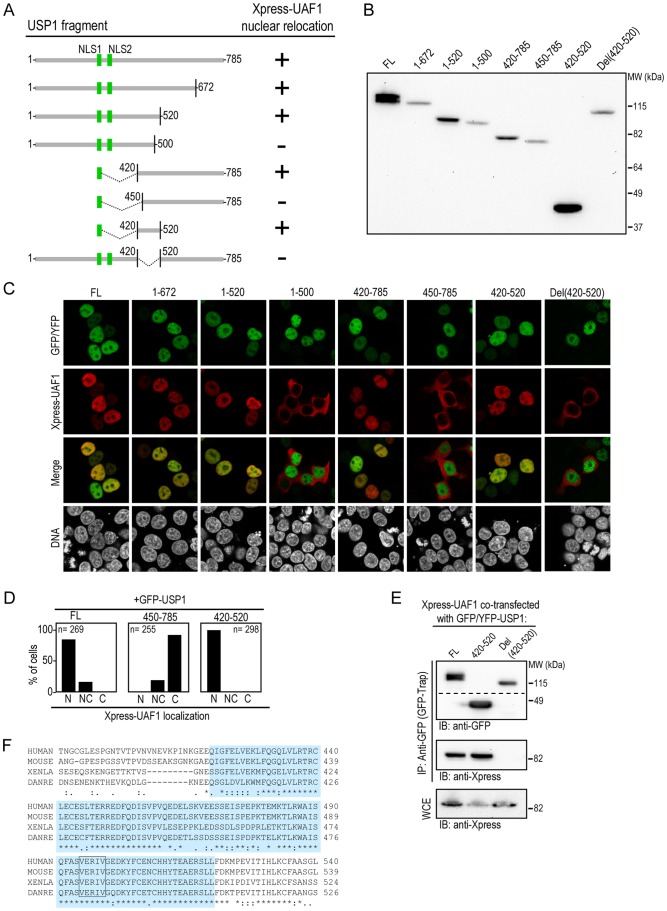
Mapping the UAF1-binding site in USP1. A. Schematic representation of USP1 deletion mutants used to map the UAF1-binding site. The critical USP1 nuclear localization signals NLS1 and NLS2 are depicted as green rectangles. The ability of each fragment to induce (+) or not (−) nuclear relocation of Xpress-UAF1 is indicated to the right. B. Immunoblot analysis demonstrating the expression and the correct size of the different USP1 mutant proteins. C. Confocal images show representative examples of 293T cells co-expressing Xpress-UAF1 (red) with full-length (FL) GFP-USP1 or with the different deletion mutants (green). Nuclear relocation of Xpress-UAF1 is induced by the fragments (1–672), (1–520), (420–785) and (420–520), but not by the fragments (1–500), (450–785), or the construct with the interstitial deletion Del(420–520). Cells were counterstained with Hoechst to show the nuclei (DNA panels). D. Semiquantitative analysis of Xpress-UAF1 nucleocytoplasmic distribution in three of the samples shown in panel C. The number of cells counted in each sample (n) is indicated within the graph. E. Co-immunoprecipitation analysis, using GFP-trap, showing that full-length USP1 and the (420–520) fragment, but not the USP1 Del(420–520) mutant, interact with Xpress-UAF1 in co-transfected 293T cells. The upper panel shows that the three USP1 proteins were efficiently pulled down by the GFP-trap reagent (the dotted line indicates that the panel is a composite of two images from the same gel). The middle panel shows that Xpress-UAF1 was co-immunoprecipitated with FL USP1 and the (420–520) fragment, but not with the Del(420–520) mutant, an observation that is entirely consistent with the results obtained in the relocation assay. The lower panel shows the expression levels of Xpress-UAF1 in the whole-cell extract (WCE) as control. F. Alignment of human USP1 protein sequence encompassing the UAF1-binding domain (blue) with USP1 proteins from mouse, *Xenopus* (XENLA) and zebrafish (DANRE). The amino acid sequence VERIV, which resembles a UAF1-interacting motif in HPV E1 protein [17], is boxed.

Leptomycin B (Apollo Scientific) was added to the culture medium 24 hours after transfection to a final concentration of 6 ng/ml for 3 hours.

### Immunofluorescence and Confocal Microscopy Analysis

Cells expressing proteins tagged with GFP or YFP were fixed with 3.7% formaldehyde in phosphate-buffered saline (PBS) for 30 min, incubated with Hoechst 33285 (Sigma) to visualize the nuclei, washed with PBS, and mounted onto microscope slides using Vectashield (Vector). Endogenous USP1 and Xpress-tagged UAF1 were stained with anti-USP1 polyclonal antibody (Abcam ab27868, 1∶200) and anti-Xpress monoclonal antibody (Invitrogen, 1∶300), respectively, using a previously described immunostaining procedure [22].

**Table 1:** Amino acid identity across several functional domains between human USP1 protein and USP1 proteins from the mouse, the clawed frog *Xenopus laevi* (xenla), and the zebrafish *Danio rerio* (danre).

	% amino acid identity (ClustalW score)		
**human USP1 domain** *	**mouse USP1**	**xenla USP1**	**danre USP1**
Overall (1–785)	88	54	49
UAF1-binding (420–520)	100	81	79
Cys (82–99)	100	100	100
Asp (197–213)	100	100	94
His (576–784)	89	48	37

* Cys, Asp and His domains as described in [10].

Slides were examined and images acquired using an Olympus Fluoview FV500 confocal microscope. Sequential acquisition of each fluorochrome was performed in order to avoid overlapping of fluorescent emission spectra.

### Immunoblot Analysis and Co-immunoprecipitation

For immunoblot analysis, protein samples were resolved in a 10% SDS-PAGE gel and transferred to a nitrocellulose membrane. Membranes were blocked with 5% non-fat dry milk diluted in TTBS for 1 hour, probed with either anti-GFP (Chromotek, 1∶1000) or anti-Xpress (Invitrogen, 1∶5000) antibodies for 1 hour at RT, and subsequently incubated with the corresponding horseradish peroxidase-conjugated secondary antibody (Amersham, 1∶3000). Finally, immunoblots were developed with ECL chemiluminiscence reagent (Thermo Scientific). Anti-GFP immunoprecipitation was carried out using the GFP-Trap_M reagent (Chromotek), following manufacturer’s directions. Immunoprecipitated proteins were analysed by immunoblot as described above.

**Figure 5:**
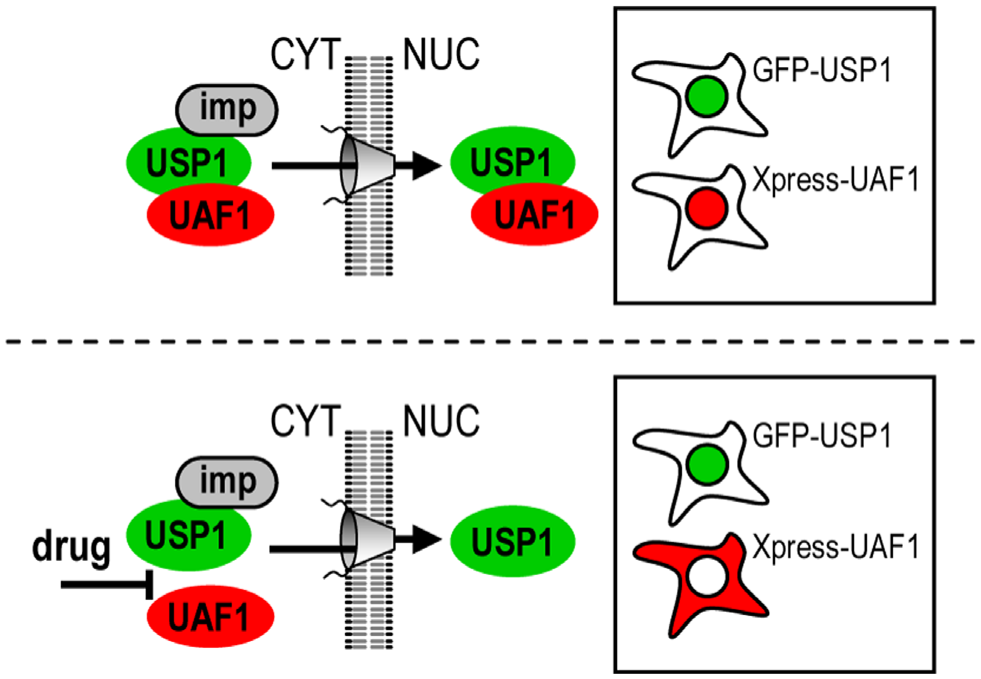
A cellular relocation assay to screen for drugs that interfere with USP1/UAF1 complex formation. In the upper part, the formation of the USP1/UAF1 complex can be assessed in cells co-transfected with GFP-USP1 and Xpress-UAF1, on the basis of the nuclear relocation of Xpress-UAF1. In the lower part, a drug that prevents USP1/UAF1 complex formation can be identified on the basis of the cytoplasmic localization of Xpress-UAF1.

### Bioinformatics analysis

Prediction of potential NLS sequences in USP1 was carried out using PSORT (http://psort.hgc.jp/) [23] and cNLS mapper (http://nls-mapper.iab.keio.ac.jp/cgi-bin/NLS_Mapper_form.cgi) [24].

Multiple sequence alignment of USP1 amino acid sequences was carried out with ClustalW2 [25].

## Results and Discussion

### Three Candidate Nuclear Localization Signals (cNLSs) in Human USP1 Amino Acid Sequence

Human USP1 is a 785 amino acid protein which, consistent with previous reports [8] localizes to the cell nucleus when expressed as a GFP-tagged protein in 293T cells (Figure 1A, left panels). In addition, immunofluorescence analysis with an anti-USP1 antibody revealed a predominanlty nuclear staining in 293T cells (Figure 1A, right panels) and HeLa cells (data not shown). We have recently reported the presence of an NES in the amino acid sequence of human USP1 [14] but, given the steady state nuclear localization of the protein, any potential nuclear export mediated by the NES appears to be effectively counteracted by rapid nuclear import. However, the USP1 sequences that mediate its import into the nucleus have not been functionally mapped. Using two different web-based NLS prediction programs, PSORT [23] and cNLS mapper [24], we examined USP1 primary amino acid sequence searching for motifs that may constitute nuclear localization signals (NLSs). Three sequences containing short stretches of basic residues were identified by the programs as candidate NLSs (cNLSs) in human USP1 (Figure 1B).

These cNLS were tested using an *in vivo* nuclear import assay in 293T cells. In this assay, we evaluate the ability of these sequences to induce the nuclear relocation of a cytoplasmic chimeric protein (NES-GFP) that contains the green fluorescent protein fused to the previously identified nuclear export sequence NET22 (Figure 1C) [14]. NET22 is a relatively strong NES, with an export assay score of 5+ over a maximum score of 9+ [21]. As positive and negative controls, the SV40 large T antigen NLS (PKKKRKV) and a mutant version of this NLS (PAAARAV) were used, respectively. As shown in Figure 1C, the three USP1 motifs tested were able to induce the nuclear relocation of NES-GFP, although to a different extent. The (14–37) fragment behaved as a very weak NLS, being able to only partially overcome NES-mediated export, and leading to an even distribution of NES-GFP in the nucleus and cytoplasm. In contrast, the (266–287) fragment induced a near-complete nuclear relocation of the NES-GFP protein, similar to the relocation afforded by the SV40 NLS. Finally, a clear, but less-marked relocation, was induced by the (298–321) fragment. Similar results were obtained when the assay was carried out in HeLa cells (data not shown). These results, therefore, identify three USP1 amino acid motifs, having different degrees of nuclear import activity, that might contribute to the nuclear localization of this protein.

### Two NLSs are Necessary and Sufficient for USP1 Nuclear Import

In order to assess the contribution of each cNLS to USP1 nuclear localization, we generated a series of USP1 deletion mutants fused to YFP (Figure 2A). The expression and size of each USP1 fragment was confirmed by immunoblot (Figure 2B), and their nucleocytoplasmic localization was evaluated in transfected 293T cells (Figure 2C). Like the full-length protein, the fragment (1–350), lacking the carboxy-terminal half of USP1, but bearing the three cNLSs, was nuclear. The fragment (1–285), lacking cNLS[298–321], also localized to the nucleus. In contrast, the fragment (1–269), which lacks both cNLS[266–287] and cNLS[298–321], localized exclusively to the cytoplasm. These results indicate that cNLS[14–37] is not sufficient to maintain nuclear localization of USP1.

Since the cytoplasmic 1–269 fragment contains the previously identified NES [14], we hypothesized that active CRM1-dependent export might contribute to its nuclear exclusion. To test this possibility, cells were treated with the specific CRM1 inhibitor leptomycin B (LMB) [26]. Indeed, inhibition of CRM1 resulted in a partial relocation of the fragment (1–269) to the nucleus (Figure 2D), suggesting that CRM1-mediated nuclear export contributes to the marked cytoplasmic localization of this USP1 fragment. The prominent nuclear localization of full-length USP1, however, suggests that either CRM1 access to USP1 NES is somehow hindered, or any eventual nuclear export of the protein is efficiently counteracted by rapid re-import into the nucleus. Further studies should clarify this issue. Altogether, our data strongly suggested that, in the physiological context of the full-length protein, the critical nucleocytoplasmic localization motifs of USP1 were cNLS[266–287] (hereafter called NLS1) and cNLS[298–321] (hereafter called NLS2). NLS1 is a classical bipartite nuclear import signal, bearing two runs of basic amino acids separated by a short linker, whereas NLS2 is a monopartite signal with a single run of basic residues [27].

In order to further delineate the role of each NLS in the context of full-length USP1, we used site-directed mutagenesis to introduce alanine substitutions of basic residues in either or both NLSs (Figure 2E). GFP-USP1^NLS1m^ was still a predominantly nuclear protein, but a fluorescent signal was also evident in the cytoplasm of some transfected cells, indicating that mutation of NLS1 reduces, but does not abrogate, USP1 nuclear import. GFP-USP1^NLS2m^, on the other hand, was exclusively located in the nucleus, like wild type USP1. Strikingly, simultaneous mutation of both NLSs completely abrogated GFP-USP1 nuclear import. Of note, GFP-USP1^NLS1/2m^ did not relocalize to the nucleus upon LMB treatment (not shown). These results suggest that both NLS1 and NLS2 contribute to USP1 import, although NLS1 appears to play a more critical role. In summary, we have identified the two nuclear localization signals that mediate USP1 nuclear import. Interestingly, a proteomic study has identified importin alpha 5 (KPNA1) as a USP1-interacting protein [28]. It seems, thus, reasonable to propose that the NLSs identified here may mediate the interaction of USP1 with this nuclear import receptor.

### USP1 NLSs Mediate Nuclear Import of the USP1/UAF1 Complex

As previously reported [16], [17], Xpress-tagged UAF1 localized to the cytoplasm of 293T cells (Figure 3A). LMB treatment did not induce its nuclear relocation, suggesting that CRM1-mediated nuclear export is not necessary to maintain the cytoplasmic localization of UAF1.

We hypothesized that the USP1 NLSs could mediate the nuclear import of the USP1/UAF1 complex. To test this possibility, we co-expressed Xpress-UAF1 with either GFP-USP1 wild type or with GFP-USP1^NLS1/2m^. As a control, Xpress-UAF1 was co-expressed with GFP. As shown in Figures 3B and 3C, Xpress-UAF1 was cytoplasmic when co-expressed with GFP, but readily relocalized to the nucleus when co-expressed with GFP-USP1. The import-deficient mutant GFP-USP1^NLS1/2m^ was unable to relocate Xpress-UAF1 to the nucleus. These results indicate that the two USP1 NLSs identified here mediate the import of the USP1/UAF1 complex into the nucleus. The cytoplasmic localization of Xpress-UAF1 in the absence of ectopically expressed USP1 suggests that endogenous levels of USP1 in 293T cells are not sufficient to relocalize overexpressed UAF1 to the nucleus.

These findings parallel the previous observation that the E1 helicase encoded by some strains of human papillomavirus (HPV), can bind and relocate UAF1 to the nucleus [17]. HPV E1-mediated nuclear relocation of UAF1 also depends on E1 NLS. Thus, UAF1 subcellular localization appears to rely on the transport signals of its different interacting partners. In this regard, it has been reported that, besides USP1, UAF1 also binds and regulates the activity of two other DUBs, namely USP12 and USP46 [29]. Thus, three different DUB/UAF1 complexes have been detected in human cells: USP1/UAF1, USP12/UAF1 and USP46/UAF1 [29]. The localization and function of USP12 and USP46 is still poorly characterized, but it has recently been shown that these enzymes deubiquitinate two nuclear proteins, namely histones H2A and H2B, in *Xenopus* [30]. It is tempting to speculate that nuclear relocation of the USP12/UAF1 and USP46/UAF1 complexes could be mediated by, as yet unidentified, NLSs in the DUBs.

Our results are consistent with a model in which USP1 and UAF1 form a complex in the cytoplasm that is subsequently translocated to the nucleus through active nuclear import mediated by the two USP1 NLSs (Figure 3D). Such a model, obviously, implies that USP1 can bind simultaneously to the nuclear import receptors and to UAF1. The region of USP1 that mediates its interaction with UAF1, however, remains to be identified.

### Mapping the UAF1-interacting Domain of Human USP1

In order to map the UAF1-binding domain in USP1 we used a cellular relocation assay based on the change that USP1 co-expression causes in the nucleocytoplasmic localization of Xpress-UAF1.

We generated a series of seven YFP- or GFP-tagged USP1 deletion mutants lacking different amino-terminal, carboxy-terminal, or interstitial amino acid segments (Figure 4A). The NLS1 sequence was added to the amino-terminal end of those USP1 fragments lacking the NLSs (420–785, 450–785 and 420–520) in order to force their nuclear localization. The expression and size of each USP1 mutant was confirmed by immunoblot (Figure 4B). Xpress-UAF1 was co-expressed with each of the USP1 deletion mutants, and the localization of both proteins was examined in transfected 293T cells. The results are summarized in Figure 4A, and illustrated in Figure 4C. As expected, all the USP1 mutants were localized in the nucleus. The fragment 1–672, mimicking USP1 autocatalytic cleavage product [10], and the fragment 1–520 induced nuclear relocation of UAF1. In contrast, UAF1 was cytoplasmic when co-expressed with the USP1 fragment 1–500, suggesting that this mutant was unable to bind UAF1. On the other hand, the USP1 fragment 420–785, but not the fragment 450–785, was able to relocate UAF1 to the nucleus. The 100 amino acid fragment 420–520 readily induced nuclear accumulation of UAF1, whereas deletion of this fragment in the mutant Del(420–520) abolished nuclear relocation of co-expressed UAF1. Combined, these results indicate that USP1 region 420–520 mediates its interaction with UAF1. In fact, a semiquantitative analysis of Xpress-UAF1 nucleocytoplasmic distribution (Figure 4D) showed that the 420–520 fragment was even more efficient than full-length USP1 in promoting nuclear accumulation of UAF1. This observation raises the possibility that UAF1 interaction may be negatively regulated by USP1 sequences outside the UAF1-binding domain.

The role of the USP1 fragment (420–520) in mediating UAF1 binding was further confirmed by co-immunoprecipitation analysis. As shown in Figure 4E, full-length USP1 and the (420–520) fragment, but not the USP1 Del(420–520) mutant, interact with Xpress-UAF1 in co-transfected 293T cells.Alignment analysis of USP1 proteins from human, mouse, frog and fish (Figure 4F and Table 1) revealed that the UAF1-binding domain is highly conserved among different vertebrate species. For example, whereas the overall amino acid identity between human and zebrafish USP1 is 49%, the identity within the UAF1-binding domain reaches 79%. Although this high degree of conservation may be due to a variety of reasons, it is entirely consistent with the possibility that the UAF1 interacting domain identified here is functionally relevant.

It has been previously shown that a short amino acid motif of HPV E1 protein (VEAIV) is critical for UAF1 binding [17]. Double amino acid substitutions in this motif (VE or IV to AA) effectively abrogate E1/UAF1 interaction. We noted the presence of a similar motif (VERIV), located at amino-acid position 495–499 within the UAF1-binding domain of USP1 (Figure 4F). However, mutation of this sequence to AARAA did not abrogate UAF1 interaction (data not shown), indicating that the VERIV motif is not essential for USP1/UAF1 complex formation.

The identification of the UAF1-binding domain in USP1 may be of clinical relevance in the light of the recent observation that interfering with the activity of the USP1/UAF1 complex sensitizes non-small cell lung cancer cells to DNA damaging agents [20]. This observation suggests that the USP1/UAF1 complex may be an important novel target in cancer treatment and thus, interfering with the formation and/or inducing the dissociation of the complex may represent a potential therapeutic strategy. In this regard, previous studies have shown that the USP1-interacting region of UAF1 encompasses a large and ill-defined region [15], which would not be easy to target. In contrast, our findings reveal a discrete UAF1-binding interface in USP1, which could be more easily targeted and thus, more relevant from a therapeutic point of view.

Importantly, the striking change in the nucleocytoplasmic localization of Xpress-UAF1 that results from GFP-USP1 co-expression provides the basis for a relocation assay to test USP1/UAF1 complex formation in cells. Here, we have used this assay to map the UAF1-binding motif in USP1, but such an assay can also be applied to evaluate the ability of different drug treatments to interfere with the formation of the USP1/UAF1 complex (Figure 5). Furthermore, by genetically tagging UAF1 with a fluorescent protein, such as RFP, and using automated microscopy procedures, the assay can be readily adapted to a high throughput format in order to allow a high content screening for molecules that interfere with USP1/UAF1 complex formation.
